# Svapnia and Sweet Quinine

**Published:** 1869-03-01

**Authors:** 


### *Svapnia and Sweet Quinine.*

These preparations (see advertising pages of The Journal) from our own trials, and the reports of our friends, we fully believe are entitled to the merits claimed for them by the manufacturer.

